# Global plastics treaty negotiations and health-care system use of plastics, WHO African Region 

**DOI:** 10.2471/BLT.26.295879

**Published:** 2026-10-01

**Authors:** Oluwaseyi Muyiwa Egbewande, Oladotun Erastus Okediji, Adedayo Samsondeen Adeboye

**Affiliations:** a Faculty of Pharmaceutical Sciences, University of Ilorin, PMB 1515, Ilorin, Kwara State, Nigeria.

Member States adopted a resolution to develop a United Nations Global Plastics Treaty at the fifth session of the Environment Assembly in March 2022. Since then, negotiations led by the Intergovernmental Negotiating Committee, convened by the United Nations Environment Programme, have sought to develop a legally binding instrument addressing plastic pollution across its life cycle, from production to disposal.^1^ However, the final set of meetings ended without an agreement, leaving countries divided over the ambition and scope of global controls, including the extent to which plastic production should be addressed.^2^

The World Health Organization (WHO) African Region faces increasing plastic pollution alongside substantial gaps in waste-management infrastructure. These challenges apply to health-care systems, where plastics serve essential functions but also produce waste. Health-care activities in the African Region are estimated to generate almost 300 000 tonnes of plastic waste annually, with only a small proportion recycled.^3^^,^^4^ Resource constraints also contribute to reliance on point-of-care testing devices and other single-use plastic medical and laboratory products.^5^ Therefore, context-specific approaches are needed that prioritize the realities of African health-care plastic use.

In Africa, limited evidence exists on health-care-specific plastic consumption and end-of-life management, which includes collection, sorting, recycling and ultimately disposal. At the same time, uneven waste-management infrastructure and restricted access to alternatives complicate efforts to reduce plastic use. This gap in health-care-specific waste monitoring and reporting hinders the generation of comparable estimates of plastic use and management across African settings.^6^ This evidence gap makes the implications of the uniform plastic controls of the global plastic treaty difficult to assess across the continent.^7^ At the same time, reducing health-care plastics cannot be approached solely as an environmental objective because plastic products provide important clinical benefits, particularly during infectious disease outbreaks.^8^

The challenge is therefore not simply the volume of plastics used, but where, why and how they are used and managed after use. While single-use plastics provide important clinical benefits by maintaining sterility and preventing cross-contamination, they increase plastic waste generation. This impact became evident during the coronavirus disease 2019 pandemic, when use of single-use personal protective equipment increased.^5^^,^^9^

End-of-life management also presents an additional challenge. These plastic wastes pose serious threats to human health and the environment, as many single-use plastics are discarded in dumpsites and uncontrolled landfills, or are openly thrown away in parks, markets and on beaches.^9^ These wastes may contribute to conditions that increase the spread of infectious diseases or serve as breeding grounds for disease vectors. In addition, some plastics used in health care may also contain chemicals of concern, including bisphenol A, phthalates and other plastic-related chemicals associated with endocrine, reproductive, developmental and metabolic effects.^10^

Implementing universal plastic bans is highly challenging because regulatory frameworks vary across African countries. Countries such as Kenya and Rwanda, for example, have introduced restrictions on general single-use plastics, with the health-care applications receiving exemptions because of safety requirements, while other countries still have no restrictions.^11^^,^^12^ These differences illustrate the difficulty of applying uniform plastic controls across health-care systems, especially in the African Region, where low-resource settings are prevalent. For instance, a measure that is feasible for a well-resourced facility with access to safe alternatives may not be so in a setting where waste-treatment capacity is unavailable. 

This evidence indicates the need for context-specific approaches to health-care plastics within the Global Plastics Treaty. Because plastics vary in their clinical importance and environmental consequences, reduction measures should not be applied uniformly across health care. Instead, restrictions should consider clinical necessity, patient safety, the availability and affordability of safer alternatives, chemical and environmental risks, and the capacity of health facilities to segregate and safely manage waste. Essential products used for infection prevention, diagnosis, treatment and emergency care should therefore be distinguished from non-essential uses that can be reduced, substituted or redesigned.

This approach is particularly relevant in low-income settings, particularly in African countries, where limited data, uneven waste-management infrastructure and constrained access to alternatives may limit the feasibility of uniform requirements. The treaty should also incorporate health-care-specific criteria into reduction measures and implementation guidance. These measures would allow plastic use to be reduced where clinically feasible while maintaining the safety and continuity of essential health-care services.
